# Single-cytosine methylation at W-boxes repels binding of WRKY transcription factors through steric hindrance

**DOI:** 10.1093/plphys/kiad069

**Published:** 2023-02-14

**Authors:** Magali Charvin, Thierry Halter, Romain Blanc-Mathieu, Pierre Barraud, Magali Aumont-Nicaise, François Parcy, Lionel Navarro

**Affiliations:** Institut de Biologie de l’Ecole Normale Supérieure (IBENS), Centre National de la Recherche Scientifique UMR8197, Institut National de la Santé et de la Recherche Médicale U1024, 75005 Paris, France; Institut de Biologie de l’Ecole Normale Supérieure (IBENS), Centre National de la Recherche Scientifique UMR8197, Institut National de la Santé et de la Recherche Médicale U1024, 75005 Paris, France; Laboratoire Physiologie Cellulaire et Végétale, Université Grenoble Alpes, CEA, CNRS, INRAE, IRIG-DBSCI-LPCV, F-38054 Grenoble, France; Expression génétique microbienne, UMR 8261, CNRS, Université Paris Cité, Institut de biologie physico-chimique, IBPC, F-75005 Paris, France; Institute for Integrative Biology of the Cell (I2BC), Université Paris-Saclay, CEA, CNRS, 91198 Gif-sur-Yvette, France; Laboratoire Physiologie Cellulaire et Végétale, Université Grenoble Alpes, CEA, CNRS, INRAE, IRIG-DBSCI-LPCV, F-38054 Grenoble, France; Institut de Biologie de l’Ecole Normale Supérieure (IBENS), Centre National de la Recherche Scientifique UMR8197, Institut National de la Santé et de la Recherche Médicale U1024, 75005 Paris, France

## Abstract

DNA methylation is an epigenetic mark that fine-tunes gene expression, notably by negatively or positively regulating transcription factor (TF)-DNA binding. In plants, DNA methylation has primarily been shown to inhibit TF-DNA binding. However, little is known about the underlying mechanisms. Here, we show that DNA methylation decreases the binding of several Arabidopsis (*Arabidopsis thaliana*) WRKY TFs to their genomic regions and their binding sites in vitro. We also provide evidence that DNA methylation at a single cytosine located in a functional core W-box motif repels DNA binding of AtWRKY40 in vitro. Using structural modelling, we further demonstrate that this cytosine interacts through van der Waals contacts with the conserved tyrosine of WRKY-DNA binding domains. Importantly, our model predicts steric hindrance when a 5-methyl group is present on this specific cytosine, thereby likely preventing tight binding of WRKY-DNA binding domains. Finally, because the WRKY motif and the residues involved in DNA contacts are conserved across Arabidopsis and rice (*Oryza sativa*) WRKY TFs, we propose that this methylation-dependent WRKY-DNA binding inhibitory mechanism could be widespread across plant species.

## Teaser

Methylation of a single cytosine in the W-box element alters DNA binding of WRKY transcription factors through steric hindrance.

## Introduction

Transcription factors (TFs) are central regulators of gene expression and control a wide range of biological processes. They can bind to specific genomic DNA sequences through recognition of Transcription Factor Binding Site (TFBS) and further activate or repress a large repertoire of genes. In eukaryotic cells, transcription is known to be regulated in the context of chromatin, whereby TFs typically compete with nucleosomes for genomic DNA accessibility (Klemm et al. 2019). Nevertheless, some TFs, referred to as “pioneer” TFs, have the ability to bind nucleosome-rich regions (Zaret et al. 2020; Lai et al. 2021; Jin et al. 2021). Besides nucleosome density, some DNA- or histone-based modifications can additionally modulate the accessibility of TFs (Klemm et al. 2019). This is notably the case of DNA methylation, an epigenetic mark that resides in the methylation of the 5-position of cytosine in DNA, also known as 5-methylcytosine or 5mC. In mammals, almost all 5mC are in the CG context, whereas in plants cytosine methylation occurs in symmetrical (CG or CHG) and in asymmetrical CHH contexts (H = A, T, or C) (Law and Jacobsen 2010). In Arabidopsis (*Arabidopsis thaliana*), methylation nearby transcriptional start sites (TSS) is generally associated with transcriptional silencing (Ando et al. 2019), suggesting that it restricts DNA/chromatin accessibility for TFs and/or components of the transcription machinery. It has been shown that DNA methylation often blocks TF-DNA binding (Yin et al. 2017; O’Malley et al. 2016). For example, a high-throughput TF-binding site discovery method, named DNA Affinity Purification sequencing (DAP-seq), reported that ∼75% of Arabidopsis TFs are sensitive to DNA methylation, meaning that methylation has an inhibitory effect on their DNA binding capacity (O’Malley et al. 2016).

We have recently shown that the Arabidopsis REPRESSOR OF SILENCING 1 (ROS1) actively demethylates promoters of a subset of defence genes to facilitate their transcriptional activation during antibacterial immunity (Halter et al. 2021). In particular, ROS1 was shown to demethylate the promoter of *RECEPTOR-LIKE PROTEIN 43* (*RLP43*) to ensure a proper transcriptional activation of this gene in response to the flagellin-derived peptide flg22 (Halter et al. 2021). Importantly, the *RLP43* promoter region subjected to ROS1-directed demethylation was shown to contain a functional W-box, the binding motif of plant-specific WRKY TFs (Halter et al. 2021; Eulgem and Somssich 2007; Birkenbihl et al. 2017). By comparing DAP-seq *versus* ampDAP-seq, in which DNA methylation were removed from the Col-0 genomic DNA by PCR amplification, AtWRKYs binding was globally enriched in the absence of methylation (O’Malley et al. 2016; Halter et al 2021). Locus-specific DAP-qPCR revealed that binding of AtWRKY18 and AtWRKY40, two well-characterized flg22-responsive AtWRKYs (Birkenbihl et al. 2017), was detected at the *RLP43* promoter using Col-0 genomic DNA but was impaired when genomic DNA from *ros1* mutants was used for this assay (Halter et al. 2021). This study thus showed that the hypermethylation at the *RLP43* promoter directly repels the binding of these AtWRKYs in vitro. However, the detailed mechanisms responsible for the repelling effect exerted by methylation at the DNA-WRKY interface, and the specific methylcytosine(s) involved in this process, remained elusive.

Here, we show that DNA methylation decreases binding of a subset of Arabidopsis WRKY TFs at their whole targeted genomic regions and at TFBS. Furthermore, we provide evidence indicating that DNA methylation of a single cytosine, located in the functional W-box element of the *RLP43* promoter, repels WRKY-DNA binding. Finally, we show that the presence of a 5-methyl group at this cytosine likely alters binding of WRKY TFs through steric hindrance. Overall, this work describes a detailed molecular mechanism by which cytosine methylation impedes binding of WRKY TFs and has important implications in the regulation of plant transcriptomes during stress responses.

## Results

### An increased number of methylated cytosines in the whole bound genomic regions and in the TFBS of AtWRKYs reduces their DNA binding affinity

**Figure 1.**
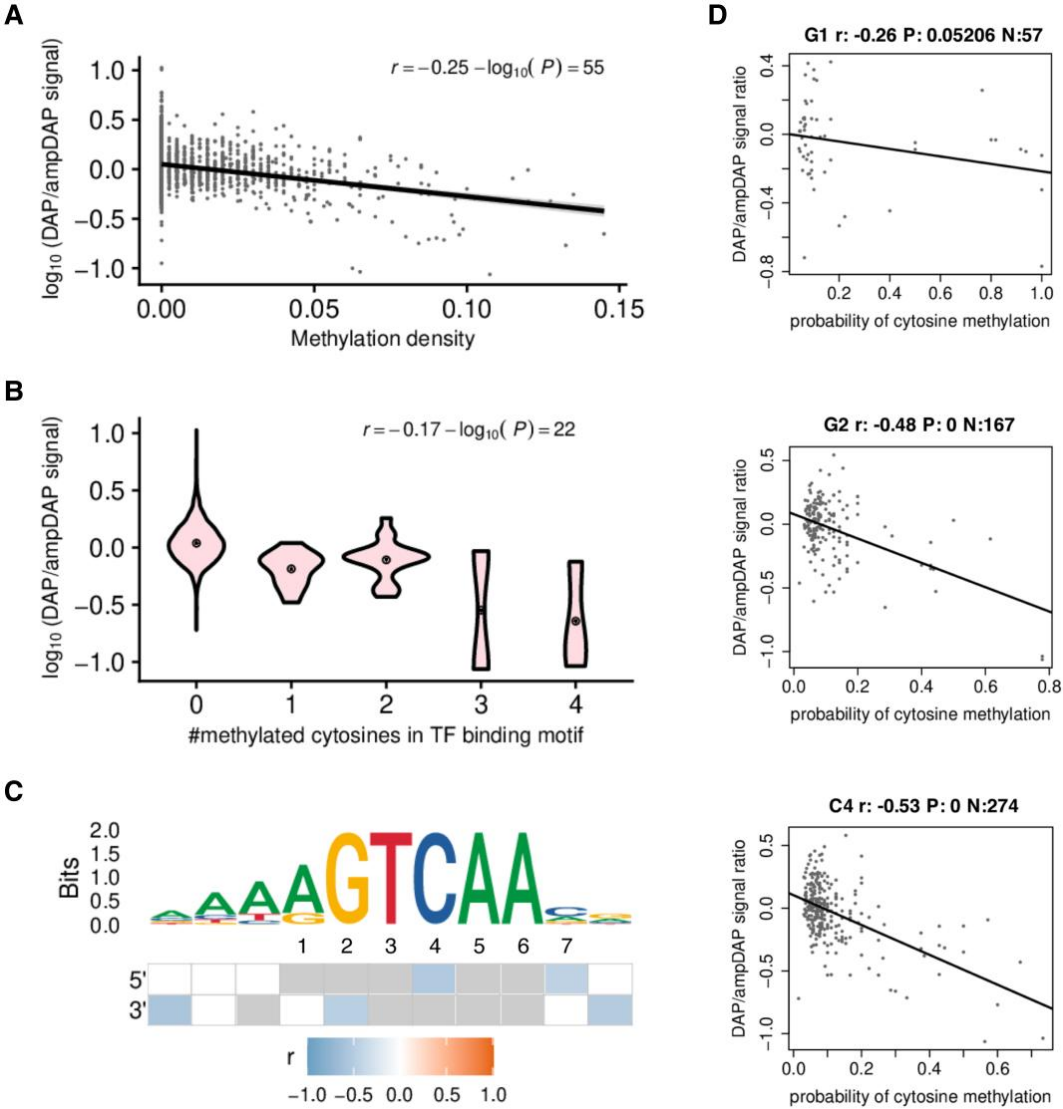
Effect of cytosine methylation on AtWRKY40-DNA binding. A) Biplot between the DNA Affinity Purification (DAP)/amplified DAP (ampDAP) signal ratio (peak normalized read coverage in the DAP experiment divided to that in the ampDAP experiment) in a log10 scale and methylation density (proportion of cytosines with a probability of methylation greater than 0.5) within TF bound regions. The increasing methylation density has a negative effect on AtWRKY40 binding. B) Violin plots of DAP/ampDAP signal ratio in a log10 scale as a function of the number of methylated cytosines in the best transcription factor (TF) binding site (TFBS) of each bound region. AtWRKY40 binding is negatively affected by the increased number of methylated cytosines. C) Binding site sequence motif and the methylation effect on each individual position. The heatmap describes the Pearson's correlation coefficient (r) between the DAP/ampDAP signal ratio in a log10 scale and the probability of methylation at each position of the best TFBSs. Blank positions have a high false discovery rate (> 5%) and grey indicates positions with less than ten cytosines in the dataset. Correlations are tested on both sides. D) Effect of methylation on individual positions at the core W-box on AtWRKY40 binding. Relation between methylation probability at a single nucleotide position in the predicted best AtWRKY40 binding site within bound regions, and the log10-scaled relative binding intensity of a DAP-seq *versus* an ampDAP-seq experiment at bound regions for AtWRKY40 at the 3 different cytosine sites. *P*-values are adjusted for multiple testing using the Benjamini and Hochberg procedure.

To get some insights into the mechanisms by which DNA methylation could inhibit AtWRKY40-DNA binding, we made use of available DAP-seq and ampDAP-seq data sets (O’Malley et al. 2016). We first plotted the DAP/ampDAP signal ratio as a function of the methylation density in the whole AtWRKY40 bound genomic regions, as previously reported (Lai et al. 2021). This analysis revealed that the DAP/ampDAP signal ratio decreased with methylation density (Fig. 1A). We further plotted the DAP/ampDAP signal ratio relative to the number of methylated cytosines within WRKY best binding sites and revealed that an increased number of methylated cytosines in the TFBS decreased AtWRKY40-DNA binding (Fig. 1B; Lai et al. 2021). A similar pattern was observed for six additional WRKYs, namely AtWRKY14, 15, 22, 24, 25 and 27 (Supplemental Fig. S1). Collectively, these data confirm previous findings indicating that methylation inhibits binding of AtWRKYs and show that this binding inhibitory effect is not only detected at the whole bound genomic regions but also at TFBS (O’Malley et al. 2016, Halter et al. 2021).

### Cytosine methylations at W-box cis-elements negatively regulate the binding affinity of AtWRKYs, with 5mC4 exhibiting the strongest inhibitory effect

We next computed the impact of individual cytosine methylation on TF-DNA binding (Lai et al. 2021, Materials & Methods). This analysis revealed that methylation at specific cytosines from the TFBS decreases DNA binding of AtWRKYs (Fig. 1, C and D; Supplemental Fig. S2). In particular, DNA methylation at the three cytosines from the W-box motif systematically impacted WRKY-DNA binding, with the most pronounced inhibitory effects detected on cytosines at position 4 on the forward strands (5mC_4_, CHH context) for six out of the seven AtWRKYs studied (Fig. 1D; Supplemental Fig. S2). 5mC_4_ and cytosine methylation at position 2 on the reverse strands (5mC_2,_ CHH or CHG contexts) exhibited strong inhibitory effects on AtWRKY40-TFBS binding (r = -0.53 and −0.48, respectively), while methylation at position 1 on the reverse strands (5mC_1,_ CHH context) showed milder negative effects (r = −0.26) (Fig. 1D). Altogether, these results suggest that methylation at cytosines embedded in the core W-box motif alters AtWRKY-DNA binding, with 5mC_4_ exhibiting the most pronounced inhibitory effect.

### DNA methylation of the cytosine at position 4 of a functional W-box severely reduces AtWRKY40-DNA binding affinity

**Figure 2.**
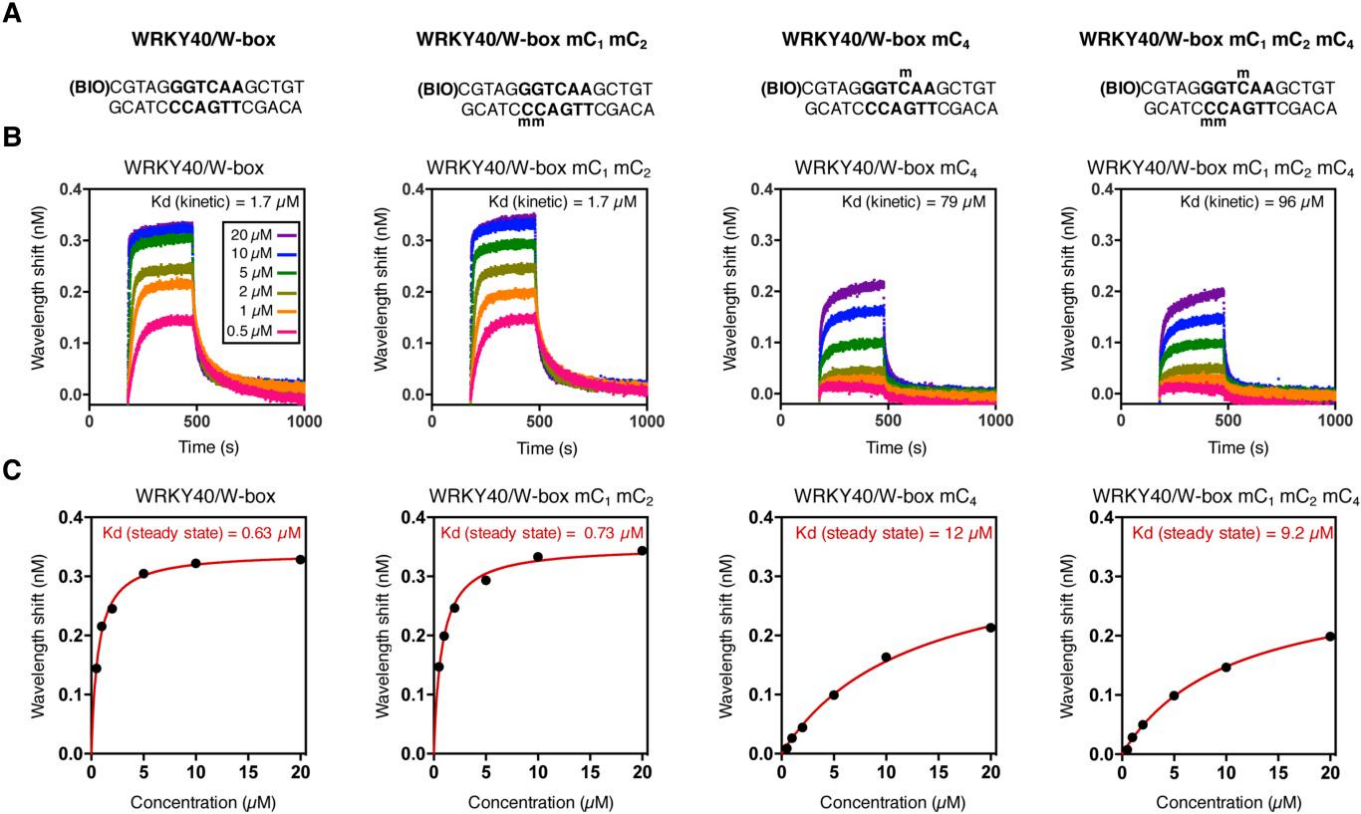
Methylation at the cytosine C4 embedded in a functional W-box has a strong negative impact on AtWRKY40-DNA binding. A) Biotinylated DNA duplexes used for the Bio-Layer Interferometry (BLI) experiments. B) Binding curves of AtWRKY40-DNA-binding domain (DBD) with DNA duplexes containing W-boxes with indicated methylation status. C) BLI-derived steady state analysis representing binding responses of AtWRKY40 DBD (nM) to DNA duplexes as a function of AtWRKY40 DBD concentration.

To understand the causal role of each cytosine methylation on WRKY-DNA binding, we used Bio-Layer Interferometry (BLI) and measured the binding capacity of AtWRKY40 at the functional W-box element of the *RLP43* promoter (Halter et al. 2021). More specifically, biotinylated double-stranded DNA duplexes corresponding to a 16-mer region of the *RLP43* promoter with specific unmethylated and methylated cytosines in the core W-box sequence were synthesized (Fig. 2A). Each DNA duplex was immobilized onto a streptavidin biosensor, and the resulting bio-layer was introduced into solutions containing purified DNA-binding domains (DBDs) of AtWRKY40 (Supplemental Fig. S3). We subsequently recorded changes in optical wavelength, which are associated with the variation in the thickness of the bio-layer resulting from the association of the DNA duplex with AtWRKY40 molecules. We found strong wavelength shifts on the bio-layer containing the unmethylated DNA duplex, a feature that was observed at the five AtWRKY40 DBD protein concentrations tested (Fig. 2B). These results demonstrate effective interactions between AtWRKY40 and the unmethylated DNA duplex, which were further supported by a constant of dissociation (K_D_) value of 630 nM at the steady state level (Fig. 2C). 5mC_1_ and 5mC_2_ (both in CHH contexts), located on the reverse strand of the W-box motif did not alter DNA binding, as wavelength shifts and K_D_ value were similar to the ones detected with the unmethylated DNA duplex (Fig. 2). By contrast, the wavelength shifts were substantially reduced, and the K_D_ value significantly higher (12 µM), when the cytosine at position 4 from the forward stand (5mC_4,_ CHH context) of the W-box motif was methylated (Fig. 2). It is noteworthy that the binding inhibitory effect triggered by 5mC_4_ was almost as strong as the one caused by a cytosine to thymine substitution, which is known to abolish WRKY-DNA binding (Supplemental Fig. S4; Ciolkowski et al. 2008). This result further supports a severe, albeit not complete, negative impact of 5mC_4_ on AtWRKY40-DNA binding. Furthermore, no additive effect on the wavelength shift, nor on the K_D_ value (9.2 µM), were found when the C1 and C2 nucleotides from the reverse strand of the W-box element were methylated besides 5mC_4_ (Fig. 2). Altogether, these data provide solid evidence that 5mC_4_ at a functional W-box element has a strong and specific inhibitory effect on the ability of AtWRKY40 to bind DNA.

### The tyrosine residue of the WRKY domains of AtWRKY40 and AtWRKY4 make van der Waals contacts with the C4 of the W-box motif

**Figure 3.**
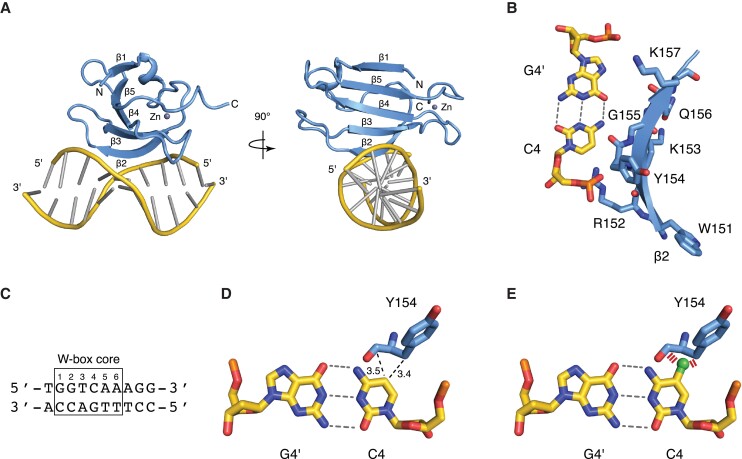
Structural model of the AtWRKY40 DBD in complex with a W-box DNA duplex. A) Lowest energy structure from the ensemble of models of the complex between AtWRKY40 and the W-box element of panel C. The protein is shown as a cartoon in light blue, the five β-strands are labelled, and the zinc ion is shown as a grey dot. The DNA backbone is shown as a cartoon in yellow and the base-pairs are shown with the ladder representation in grey. B) Close-up view of the WRKY motif of β2 (W151-K157). The protein is shown in light blue with the side chains represented as sticks. The C4-G4′ DNA base-pair is shown as sticks in yellow. The β2 strand enters deeply into the DNA major groove at the level of the C4-G4′ base-pair. In particular, the aromatic ring of C4 makes van der Waals contacts with the side-chain of Y154. C) Sequence of the DNA W-box element used for modelling the interaction of AtWRKY40 with DNA. D) The position 5 of C4 makes van der Waals contacts with Y154. E) Modelling a methyl group (in green) onto unmodified C4 from the W-box core reveals steric hindrance (indicated with red strips) with Y154.

To further understand the detailed mechanism by which 5mC_4_ from the W-box element repels AtWRKY40-DNA binding, we built a structural model of the AtWRKY40 DBD in complex with a W-box DNA duplex. For this end, we first generated a structural model of the WRKY domain of AtWRKY40 (residues 131–207) using homology modelling with Swiss-Model (Waterhouse et al. 2018; Supplemental Fig. S5, A–C). We then built the protein–DNA complex model, by superimposing the AtWRKY40 homology model onto the nuclear magnetic resonance (NMR) structural ensemble of the C-terminal WRKY domain of AtWYRK4, in complex with a W-box DNA element (PDB code 2lex) (Yamasaki et al. 2012). The resulting bundle of structures was refined with a restrained simulated annealing protocol. The ten lowest energy structures of the protein–DNA complex were pooled (Supplemental Fig. S5, D and E) and protein–DNA contacts were carefully analyzed in this bundle (Fig. 3). We noticed that in all structures of the bundle, the aromatic ring of C4 makes van der Waals contacts with the side-chain of Y154 (Fig. 3, B and D). We also analyzed the interface between AtWRKY4 and the W-box DNA and found that the binding mode of AtWRKY4 also involves van der Waals contacts between the aromatic ring of C4 in the W-box and the Y417 residue, the equivalent of Y154 in AtWRKY40 (Yamasaki et al. 2012; Supplemental Fig. S6). These analyses therefore unveiled the presence of van der Waals interactions between the tyrosine residues of the WRKY domains of two Arabidopsis WRKYs and the C4 of the W-box DNA.

### The interaction between the tyrosine residues of the WRKY domains of AtWRKY40 and 4 and the C4 of the W-box is incompatible with a 5-methyl group at this nucleotide

We next modelled the impact of 5mC_4_ on the binding of the C4 nucleotide to the Y154 and Y417 residues of AtWRKY40 and AtWRKY4, respectively. Importantly, our model predicts that the presence of a 5-methyl group at this specific cytosine would prevent the β2-strand of the DNA-binding domains of both AtWRKY TFs to deeply enter into the DNA major groove at the level of the W-box element (Fig. 3A; Supplemental Fig. S6). This interaction mode is therefore incompatible with the presence of a 5-methyl group on this cytosine (Fig. 3E; Supplemental Fig. S6). In addition, we found that both the WRKY motif in β2, and the residues involved in DNA contacts, are well conserved among different WRKY domains from Arabidopsis and rice WRKY TFs (Supplemental Fig. S7). The steric hindrance preventing optimal W-box DNA-binding by AtWRKY40 and 4 in the presence of a 5-methyl group (Fig. 3E; Supplemental Fig. S6) is thus likely a feature shared by several WRKY domains. Overall, our model suggests that 5mC_4_ of the W-box element repels binding of WRKY TFs by preventing the occurrence of van der Waals interactions between the C4 nucleotide of the W-box and the conserved tyrosine residue of WRKY-DNA binding domains.

## Discussion

WRKY TFs are known to bind to the core W-box element (TTGAC/T or G/ATCAA), which represents the consensus motif required for specific DNA binding (Eulgem and Somssich 2007). Importantly, each residue of the WRKYGQK domain is highly conserved and plays a critical role in WRKY-DNA binding (Maeo et al. 2001; Duan et al. 2007; Ciolkowski et al. 2008; Chen et al. 2019). Here, we have conducted an in-depth characterization of the impact that cytosine methylation could have on WRKY-DNA binding. Using DAP/ampDAP-seq data sets, we showed that the methylation density inhibits the binding of seven Arabidopsis WRKYs on their targeted genomic regions. Furthermore, we showed that an increased number of methylated cytosines at TFBS negatively regulates the binding of these AtWRKYs. In particular, methylation of cytosines located in the core W-box element contributed to the DNA binding inhibitory effect, with 5mC_4_ exhibiting the most pronounced repelling effect. By using BLI, we further demonstrated that 5mC_4_ at a functional W-box element severely reduced the ability of AtWRKY40 to bind DNA. Using structural modelling, we showed that the cytosine at position 4 of the core W-box element makes van der Waals contacts with the tyrosine residue of the WRKY domain of AtWRKY40. In addition, our model predicts that the negative effect triggered by 5mC_4_ at the W-box element is caused by steric hindrance, which likely prevents the β2-strand of AtWRKY40 WRKY domain to deeply enter into the DNA major groove at the level of the W-box element, thereby preventing tight binding to DNA. This prediction was also true when we analyzed the interface between AtWRKY4 and the W-box DNA. These data suggest that steric hindrance caused by cytosine methylation is a general phenomenon that does not only dampen DNA binding of human TFs, as previously shown (Yin et al. 2017, Héberlé and Bardet 2019), but also of plant TFs (Wang et al. 2020; this study). Finally, we found that both the WRKY motifs in β2, and residues involved in DNA contacts, were conserved among the WRKY domains of Arabidopsis and rice WRKY TFs. We, therefore, propose that this methylation-dependent WRKY-DNA binding inhibitory mechanism could be widespread across plant species.

## Materials and methods

### Genome wide effects of methylated cytosines on WRKY-DNA binding

To assess the effect of cytosine methylation on the WRKY binding, we compared the binding intensity of AtWRKYs in a DNA Affinity Purification sequencing (DAP-seq) experiment and in an amplified DAP-seq (ampDAP-seq) experiment in which methylation marks are erased during PCR-based amplification (Supplemental Table S1; O’Malley et al. 2016). We tested the association between the DAP/ampDAP signal ratio and the methylation levels at all bound regions (GEO accession is GSM1876327), at the best TF-binding site (TFBS) in the bound region, and at each position of the TFBS as previously described (Zhang et al. 2016; Lai et al 2021). In brief, TFBSs were searched in bound regions using a position weight matrix (PWM) constructed for each WRKYs using MEME (Machanick and Bailey 2011). The probability of cytosines methylation was taken from Zhang et al. 2016 (GEO accession is GSM1876327). Methylation density (the number of methylated cytosines in a bound region) was defined as the number of cytosines with a probability of methylation greater than 50%. Association between the relative binding intensity and methylation levels was assessed using Pearson's correlation test from R package “stats” with Benjamini-Hochberg correction for multiple tests.

### Production of recombinant AtWRKY40 DBD

DBD AtWRKY40 was cloned in the pET28a destination vector that carries a 6His-tag in *N*-terminal following classical digestion/ligation with NdeI and XhoI enzymes and T4 DNA ligase and expressed in the *Escherichia coli* strain BL21 (DE3) codon plus (ThermoFisher, EC0114) in Terrific broth (TB) (Supplemental Table S2). Cultures were grown at 37°C until they reached an OD600 = 2 and protein production was induced with 1 mM IPTG, followed by overnight growth at 18°C. Bacterial cells expressing WRKY domain of AtWRKY40 were collected by centrifugation, resuspended in Lysis Buffer (1.5 × PBS, 1 mM MgAc_2_, 0,1% (v/v) NP-40, 20 mM imidazole, 10% glycerol), and lysed by sonication during 4 min on ice. Lysate was clarified by high-speed centrifugation (18,000 rpm) and then purified on 250 µl of Ni-NTA resin (Thermo Fisher Scientific, 88221). Proteins were eluted in Elution Buffer (1.5 × PBS, 1 mM MgAc_2_, 0,1% NP-40, 150 mM imidazole, 10% glycerol) and excess imidazole was removed by overnight dialysis using Spectrum™ Labs Spectra/Por™ 2 12–14 kD MWCO (FisherScientific, 15310762) into Dialysis Buffer (1.5 × PBS, 1 mM MgAc_2_, 10% glycerol, 2 mM DTT). Protein extracts recovered at different steps of the purification procedure were resolved by SDS-PAGE on a 15% acrylamide gel, which was stained with Coomassie blue. A band above 15 kDa, corresponding to AtWRKY40-DNA-Binding Domain (DBD), was clearly visible (Supplemental Fig. S3).

### Measurement of AtWRKY40-DNA interaction by BLI

BLI experiments were conducted using a FortéBio's Octet® RED96e system (Sartorius) and Streptavidin (SA) Biosensors and performed at 25 °C under 1000 rpm stirring. Biotinylated oligonucleotide (50 µM) were annealed to its non-biotinylated reverse complementary oligonucleotide (60 µM) in an Hepes-NaCl buffer (Supplemental Table S2). DNA duplexes at 40 nM were immobilized in buffer onto the surface of the SA biosensor through a cycle of Baseline (120 s), Loading (300 s), and Baseline (120 s). The DBD of AtWRKY40 was diluted to the corresponding concentrations in running buffer (50 mM Hepes pH7, 150 mM NaCl, 0.05% (v/v) Tween20) and protein–DNA interactions were monitored during 300 s. Dissociation kinetics was then followed for 900 s. Data were analyzed using FortéBio Data Analysis 12.2 (Sartorius, FortéBio®) and fitted into a 1:1 binding model from which K_on_ and K_off_ values were obtained, and equilibrium dissociation constant K_D_ values were calculated. The experiments were performed three times with similar results.

### Modelling the interaction with the W-box DNA

A homology model of the WRKY domain of AtWRKY40 was generated, using the C-terminal WRKY domain of AtWRKY1 as a template (PDB code 2ayd) and superimposed on the backbone atoms with each of the 20 refined structures of AtWRKY4-C in complex with a W-box DNA element (PDB code 2lex) (Waterhouse et al. 2018; Duan et al. 2007; Yamasaki et al. 2012). This initial superposition allowed us to generate 400 starting structures of protein/DNA complexes between AtWRKY40 and the W-box DNA, each of them built as a unique pair of conformers. Each individual structure was subjected to a refinement protocol with no experimental energy terms in CNS 1.21 (Brunger 2007), following previously described procedures (Barraud et al. 2014; Barraud et al. 2012). First, the structures were energy minimized with a conjugate gradient minimization, and subsequently a rigid body minimization with two rigid groups defined as one for the protein and one for the DNA. Second, these minimized structures were subjected to a restrained simulated annealing protocol in implicit water. It consisted of 6 ps of dynamics at 1000 K followed by cooling to 25 K over 26 ps. Different types of restraint were applied for the interface and for the rest of the molecules; (i) the side chains of beta-strands 2 and 3 (152–157 and 166–170) were set to unrestrained atoms; (ii) the backbone of beta-strands 2 and 3 were harmonically restrained to their initial position, allowing small motions for these parts; (iii) all the rest of the protein and the DNA were set to fixed atoms. The resulting complexes were finally energy minimized and the 10 best energy structures were pooled as the refined ensemble and analyzed with PYMOL.

### Accession numbers

Sequence data from this article can be found in the GenBank/EMBL data libraries under accession numbers presented in Supplemental Table S1. DNA methylation datasets can be found under GEO accession GSM1876327 (Zhang et al. 2016). DAP- and ampDAP-seq data sets are listed in Supplemental Table S1.

## Supplemental data

The following materials are available in the online version of this article.

**Supplemental Figure S1**. Effect of methylation on the DNA binding of AtWRKYs.

**Supplemental Figure S2**. Effect of methylation at each cytosine in the core W-box elements on the DNA binding of AtWRKYs.

**Supplemental Figure S3**. Purification of WRKY40 WRKY domain.

**Supplemental Figure S4**. The DNA binding inhibitory effect detected in the presence of 5mC_4_ is almost as strong as the one observed with a point mutation at this specific cytosine.

**Supplemental Figure S5**. Modelling the interaction of AtWRKY40 with a W-box element.

**Supplemental Figure S6**. Analysis of the interaction of AtWRKY4 with a W-box element.

**Supplemental Figure S7**. Residues involved in DNA contacts are conserved among different WRKY domains.

**Supplemental Table S1**. Accessions numbers of datasets and sequence data used in this study

**Supplemental Table S2**. DNA oligonucleotides used in this study.

## Supplementary Material

kiad069_Supplementary_Data
