# Supplementary material for: Single-cytosine methylation at W-boxes repels binding of WRKY transcription factors through steric hindrance
Source: Plant Physiol. 2023 Feb 14;192(1):77–84. doi: 10.1093/plphys/kiad069 (PMC10152670; doi:10.1093/plphys/kiad069)
Supplement: kiad069_Supplementary_Data [file kiad069_supplementary_data.pdf]

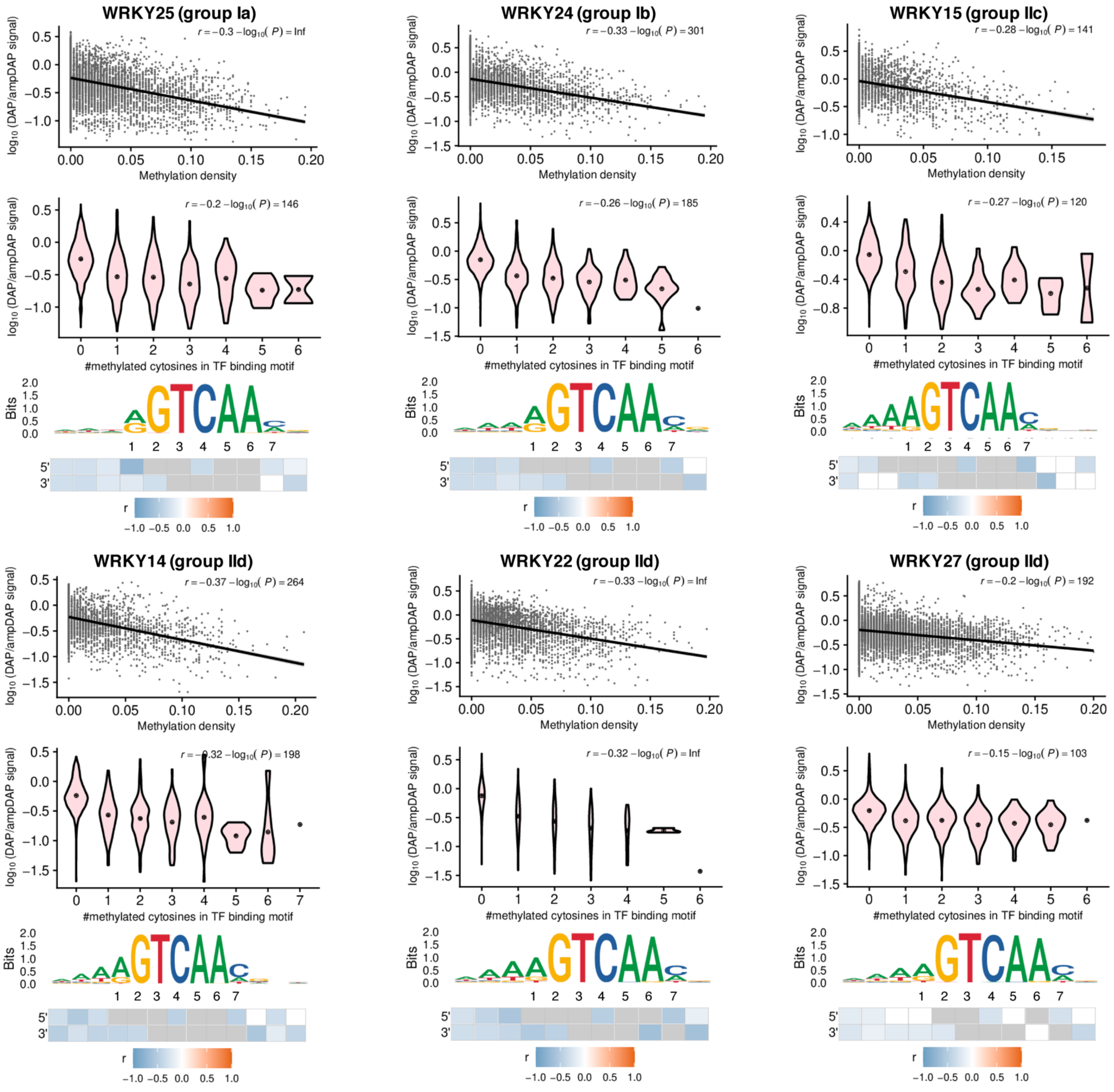

Supplemental Figure S1. Effect of methylation on the DNA binding of AtWRKYs. The impact of methylation density (upper panel), of the number of methylated cytosines (middle panel) on WRKY binding and individual cytosines (lower panel) was tested for several AtWRKY transcription factors (TFs) .

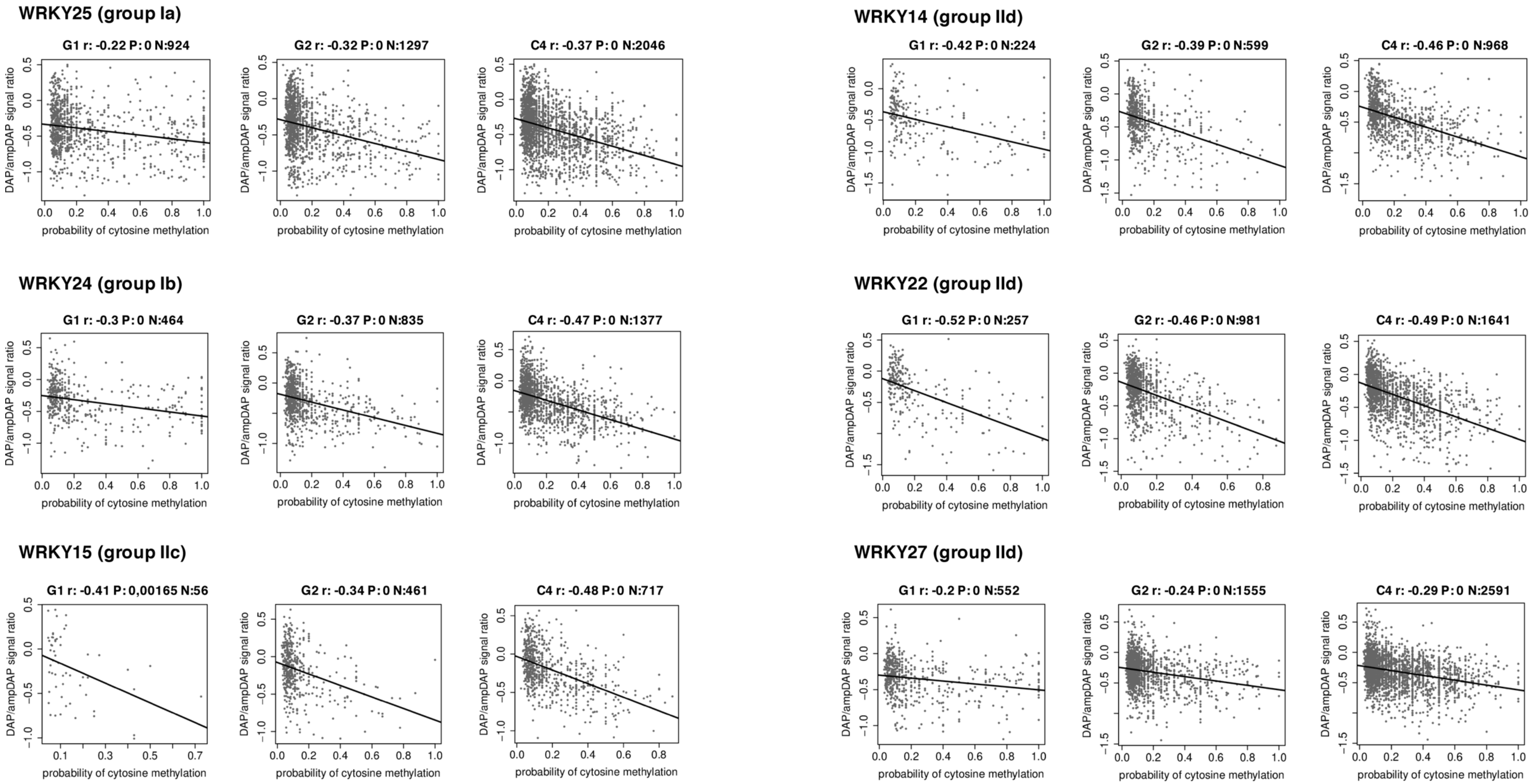

Supplemental Figure S2. Effect of methylation at each cytosine in the core W-box elements on the DNA binding of AtWRKYs. Effect of methylation on individual positions at the core W-box on the binding of each indicated AtWRKY. Relation between methylation probability at a single nucleotide position in the best predicted WRKY-binding site within bound regions, and the log10-scaled relative binding intensity of a DNA Affinity Purification (DAP)-seq *versus* an ampDAP-seq experiment at bound regions for indicated WRKYs at the 3 different cytosine sites. P-values are adjusted for multiple testing using the Benjamini and Hochberg procedure.

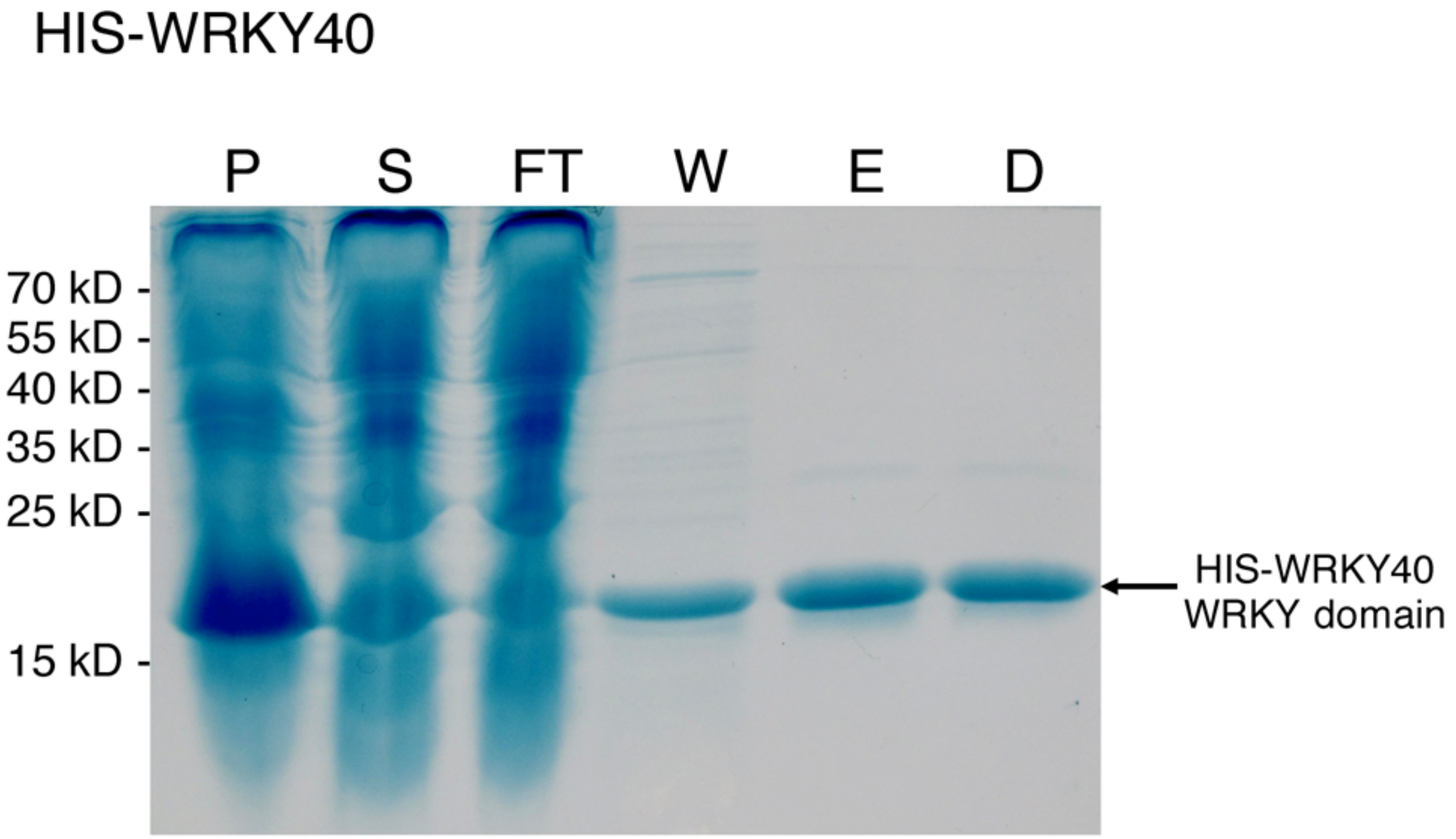

Supplemental Figure S3. Purification of WRKY40 WRKY domain. Coomassie-blue stained gel showing protein content at different steps of WRKY40 WRKY domain purification. P: Pellet after sonication; S: Supernatant; FT: Supernatant after incubation with Ni-NTA beads; W: Supernatant after washing; E: Elution; D: Elution after dialysis. An arrow indicates the His-WRKY40 WRKY domain.

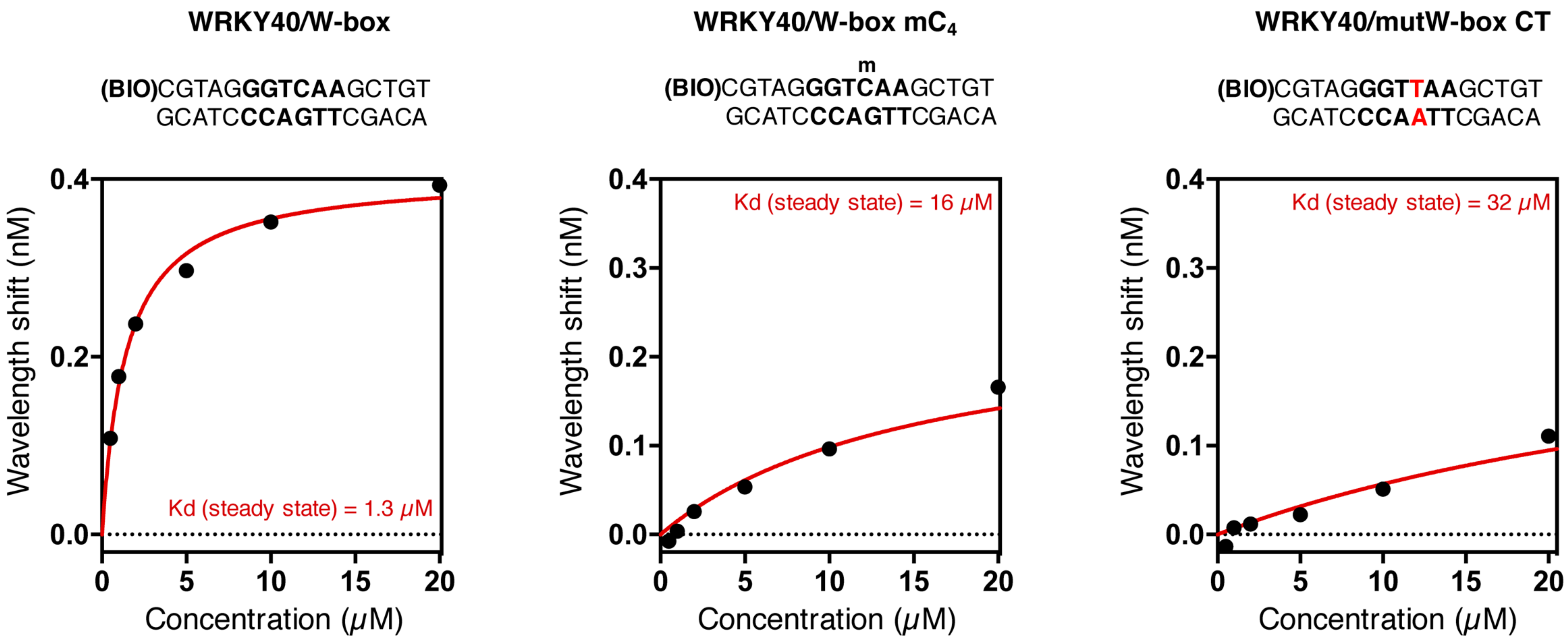

Supplemental Figure S4. The DNA binding inhibitory effect detected in the presence of $5mC_4$ is almost as strong as the one observed with a point mutation at this specific cytosine. Biolayer Interferometry **(**BLI)-derived steady state analysis representing binding responses of AtWRKY40 DNA-Binding Domain (DBD) (nM) to DNA duplexes as a function of AtWRKY40 DBD concentration.

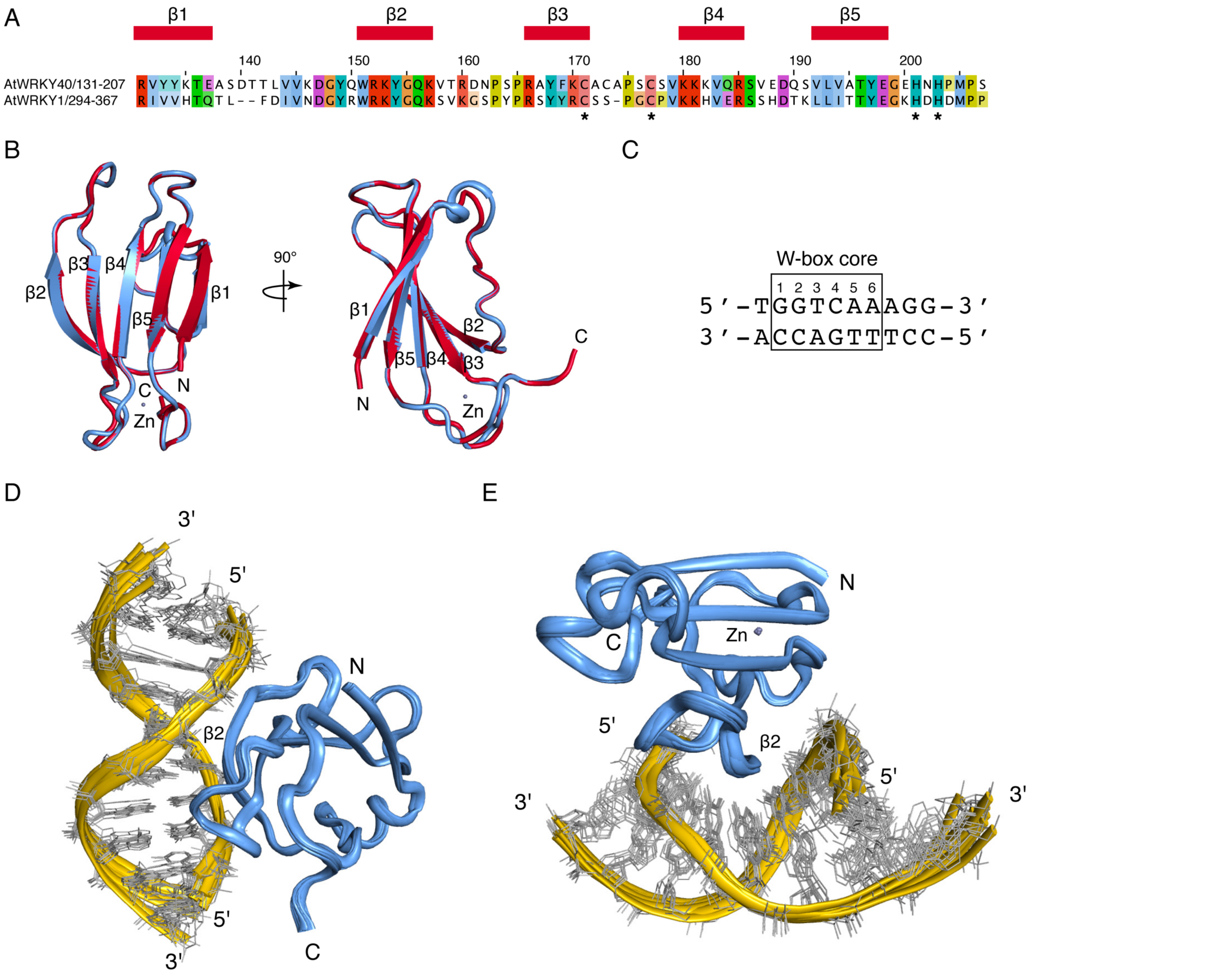

Supplemental Figure S5. Modelling the interaction of AtWRKY40 with a W-box element. (A) Sequence alignment of the WRKY domain of AtWRKY40 with the C-terminal WRKY domain of AtWRKY1. The alignment is colored by amino acid conservation and properties. The two domains share 47% of sequence identity. Residue numbering corresponds to that of AtWRKY40. The secondary structure elements of AtWRKY1 (PDB code 2ayd; Duan et al., 2007) are shown above the alignment. The CCHH zinc-binding motif is indicated by stars below the alignment. (B) Superposition of the C-terminal WRKY domain of AtWRKY1 (in red) with the homology model of the WRKY domain of AtWRKY40 (in light blue). Protein domains are show as cartoons and the five β-strands are labelled. Zinc ions are shown as grey dots. (C) Sequence of the DNA W-box element used for modelling the interaction of AtWRKY40 with DNA. This element corresponds to the W-box element in the structure of the C-terminal WRKY domain of AtWRKY4 in complex with DNA (PDB code 2lex) (Yamasaki et al., 2012). (D, E) Overlay of the 10 lowest energy structures of the AtWRKY40/W-box element model shown in two orientations. The protein is shown as a ribbon in light blue. The DNA is shown in yellow and grey.

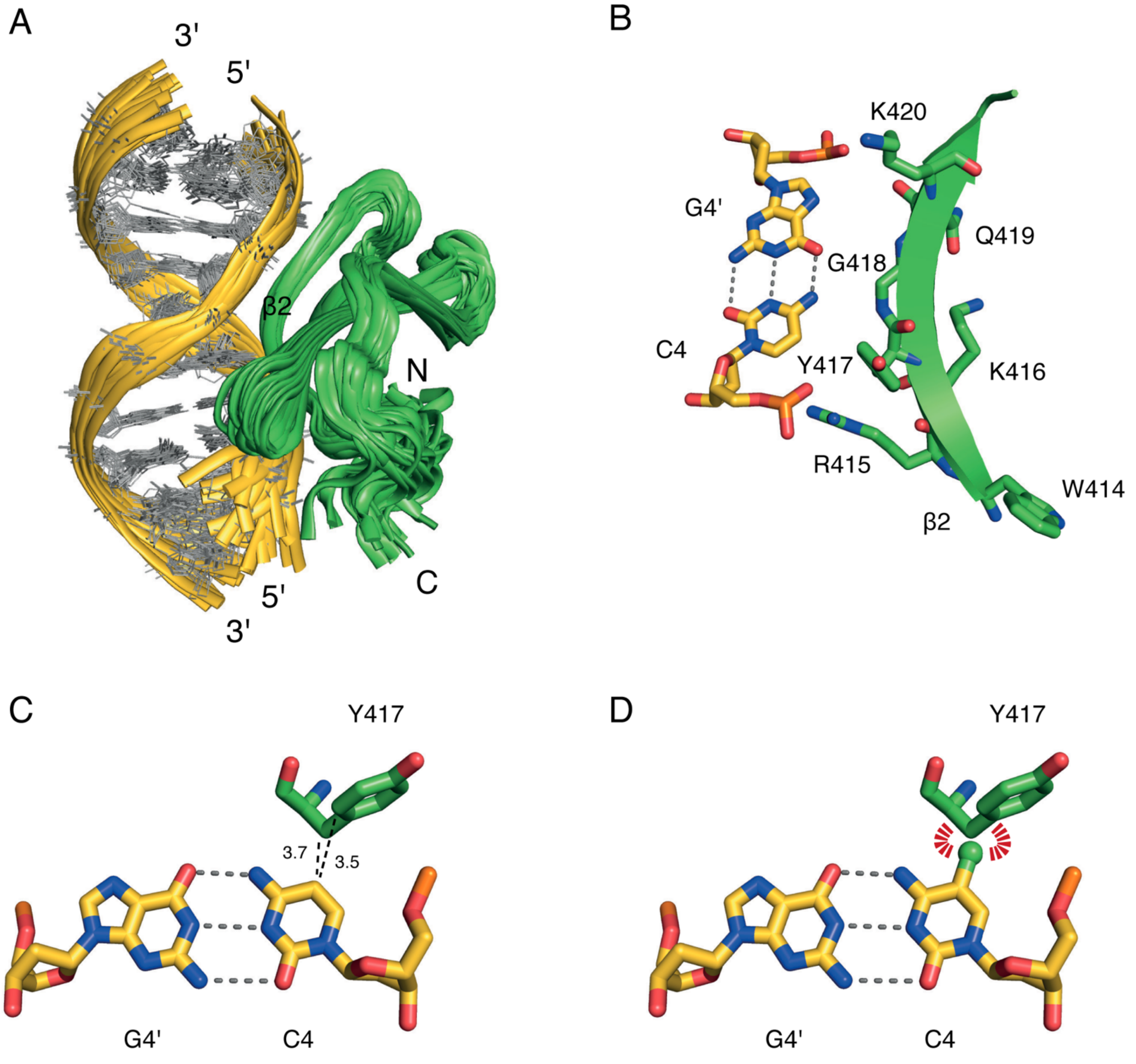

Supplemental Figure S6. Analysis of the interaction of AtWRKY4 with a W-box element. (A) NMR ensemble of the AtWRKY4/W-box element structure (PDB code 2lex) (Yamasaki et al., 2012). The protein is shown as a ribbon in green. The DNA is shown in yellow and grey. (B) Close-up view of the WRKY motif of β2 (W414-K420). The protein is shown in green with the side chains represented as sticks. The C4-G4' DNA base-pair is shown as sticks in yellow. The β2 strand enters deeply into the DNA major groove at the level of the C4-G4' base-pair. In particular, the aromatic ring of C4 makes van der Waals contacts with the side-chain of Y417. (C) The position 5 of C4 makes van der Waals contacts with Y417. (D) Modelling a methyl group (in green) onto unmodified C4 from the W-box core reveals steric hindrance (indicated with red strips) with Y417.

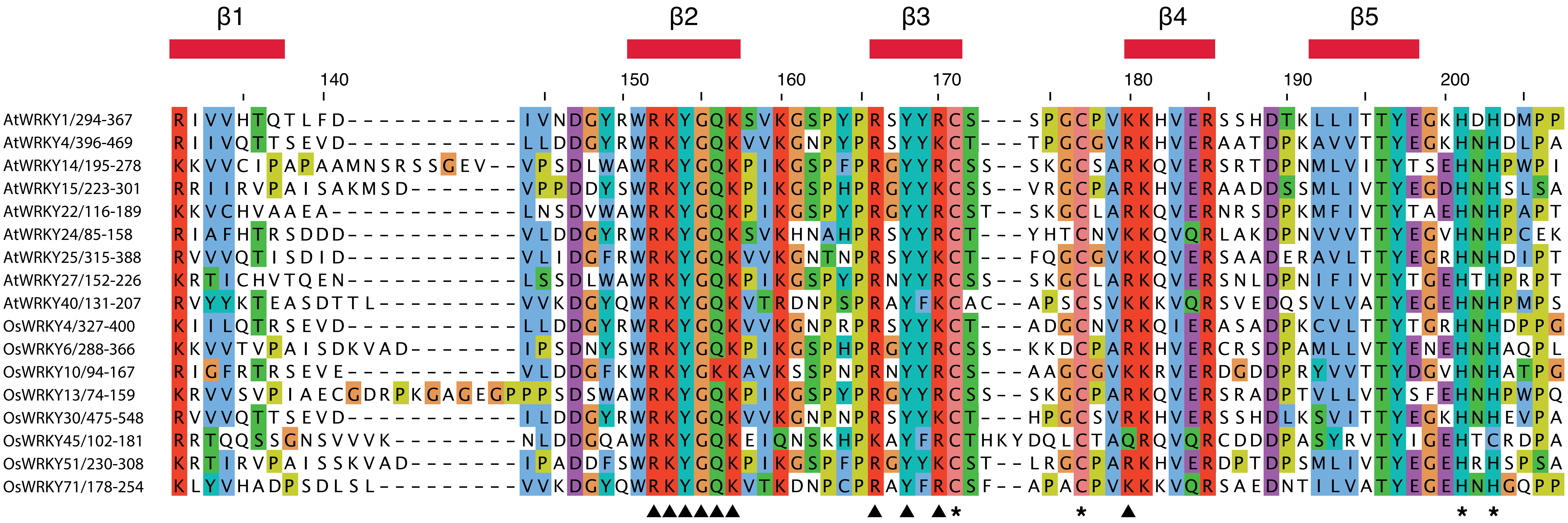

Supplemental Figure S7. Residues involved in DNA contacts are conserved among different WRKY domains. Sequence alignment of different WRKY domains, namely WRKY domains of AtWRKY1, AtWRKY4, AtWRKY14, AtWRKY15, AtWRKY22, AtWRKY24, AtWRKY25, AtWRKY27, AtWRKY40, OsWRKY4, OsWRKY6, OsWRKY10, OsWRKY13, OsWRKY30, OsWRKY45, OsWRKY51 and OsWRKY71. The alignment is colored by amino acid conservation and properties. Residue numbering corresponds to that of AtWRKY40. The secondary structure elements of AtWRKY1 (PDB code 2ayd) (Duan et al., 2007) are shown above the alignment. The CCHH zinc-binding motif is indicated by stars below the alignment. Residues involved in DNA contacts in both the NMR structure of AtWRKY4 in complex with DNA (PDB code 2lex) and our structural model of AtWRKY40 in complex with DNA (Figure 3) are indicated by arrows below the alignment.

Datasets accessions from O'Malley et al., 2016

TF	DAP/ampDAP type	GEO accessions	SRR accession
WRKY22	ampDAP	GSM1925693	SRR2926763
WRKY22	DAP	GSM1925902	SRR2926972
WRKY22	DAP	GSM1925903	SRR2926973
WRKY24	ampDAP	GSM1925694	SRR2926764
WRKY24	DAP	GSM1925695	SRR2926765
WRKY25	ampDAP	GSM1925696	SRR2926766
WRKY25	DAP	GSM1925697	SRR2926767
WRKY27	ampDAP	GSM1925700	SRR2926770
WRKY27	DAP	GSM1925701	SRR2926771
WRKY14	ampDAP	GSM1925683	SRR2926753
WRKY14	DAP	GSM1925684	SRR2926754
WRKY15	ampDAP	GSM1925685	SRR2926755
WRKY15	DAP	GSM1925686	SRR2926756
WRKY40	ampDAP	GSM1925712	SRR2926782
WRKY40	DAP	GSM1925904	SRR2926974

Sequence data from GenBank and UniProt

Gene names	Locus tags	GenBank ID	UniProtKB/Swiss-Prot accessions
RLP43	AT3G28890	822523	Q9LJW7
WRKY22	AT4G01250	827896	O04609
WRKY24	AT5G41570	834159	Q9FFS3
WRKY25	AT2G30250	817575	O22921
WRKY27	AT5G52830	835360	Q9FLX8
WRKY14	AT1G30650	839945	Q96260
WRKY15	AT2G23320	816864	O22176
WRKY40	AT1G80840	844423	Q8L9J5

Supplemental Table S1. Accessions numbers of datasets and sequence data used in this study

Oligonucleotide description	Sequence
WRKY40 DBD cloning F NdeI	CATATGAAGCAGAGAGAAGAGACTGTC
WRKY40 DBD cloning R XhoI	CTCGAGCTAATCGATCTGCGATGGCATTG
BLI primer Wbox For	(BIO)CGTAGGGTCAAGCTGT
BLI primer Wbox Rev	GCATCCCAGTTCGACA
BLI primer Wbox-mC1C2 Rev	GCATCmCmCAGTTCGACA
BLI primer Wbox-mC5 For	(BIO)CGTAGGGTmCAAGCTGT

Supplemental Table S2. DNA oligonucleotides used in this study
